# Reproductive life stages and cognitive outcomes: a systematic review in Latin American and Caribbean women

**DOI:** 10.3389/fnagi.2026.1826530

**Published:** 2026-09-17

**Authors:** Nickole Marín-Díaz, Sofia Binetti, Jaime Cañas, Carolina Panesso, Javier Villacreses, Paulina Orellana, Carolina Gonzalez-Silva, Josefina Cruzat, Joaquín Migeot, Paola Murgas, Hernán Hernández, Martin Bruno, Nilton Custodio, Rosa Montesinos, José Alberto Avila-Funes, Diana Matallana, Hernando Santamaria-García, Natalia Vilor-Tejedor, Federica Anastasi, Shireen Sindi, Andrea Slachevsky, J. E. Blümel, Sidra Sohail, Melissa Marquez, Grace E. Bardwick, Ana W. Capuano, Agustín Ibañez, Francesca R. Farina, Claudia Duran-Aniotz

**Affiliations:** 1 Latin American Brain Health Institute (BrainLat), Universidad Adolfo Ibáñez, Santiago, Chile; 2 Center for Social and Cognitive Neuroscience (CSCN), School of Psychology, Universidad Adolfo Ibáñez, Santiago, Chile; 3 Global Brain Health Institute (GBHI), Trinity College Dublin (TCD), Dublin, Ireland; 4 Escuela de Medicina, Facultad de Medicina, Universidad San Sebastián, Puerto Montt, Chile; 5 Instituto de Ciencias Biomédicas, Universidad Católica de Cuyo, San Juan, Argentina; 6 Consejo Nacional de Investigaciones Científicas y Técnicas (CONICET), Buenos Aires, Argentina; 7 Unidad de Investigación de Deterioro Cognitivo y Prevención de Demencia, Instituto Peruano de Neurociencias, Lima, Peru; 8 Unidad de Investigación y Docencia, Equilibria, Lima, Peru; 9 Centro de Investigaciones sobre el Envejecimiento, Universidad de San Martín de Porres, Lima, Peru; 10 Dirección de Enseñanza, Instituto Nacional de Ciencias Médicas y Nutrición Salvador Zubirán, Mexico City, Mexico; 11 Inserm, Bordeaux Population Health Research Center, UMR 1219, Univ. Bordeaux, Bordeaux, France; 12 Departamento de Salud Mental, Hospital Universitario Fundación Santa Fe, Bogotá, Colombia; 13 Centro de Memoria y Cognición, Hospital Universitario San Ignacio, Bogotá, Colombia; 14 Pontificia Universidad Javeriana, Bogotá, Colombia; 15 Hospital Universitario San Ignacio, Center for Brain and Cognition, Intellectus, Bogotá, Colombia; 16 Universidad Nacional de Colombia, Bogotá, Colombia; 17 Universidad de los Andes, Bogotá, Colombia; 18 Institute for Risk Assessment and Sciences (IRAS), Utrecht University, Utrecht, Netherlands; 19 Barcelona βeta Brain Research Center (BBRC), Pasqual Maragall Foundation, Barcelona, Spain; 20 Department of Human Genetics, Radboud UMC, Nijmegen, Netherlands; 21 Hospital del Mar Research Institute, Barcelona, Spain; 22 Centre for Genomic Regulation (CRG), Barcelona Institute of Science and Technology (BIST), Barcelona, Spain; 23 Division of Clinical Geriatrics, Center for Alzheimer Research, Karolinska Institute, Stockholm, Sweden; 24 Ageing Epidemiology Research Unit, School of Public Health, Imperial College London, London, United Kingdom; 25 Memory and Neuropsychiatric Center (CMYN), Neurology Department, Hospital del Salvador and Faculty of Medicine, Universidad de Chile, Santiago, Chile; 26 Geroscience Center for Brain Health and Metabolism (GERO), Santiago, Chile; 27 Departamento de Neurología y Psiquiatría, Clínica Alemana-Universidad del Desarrollo, Santiago, Chile; 28 Neuropsychology and Clinical Neuroscience Laboratory (LANNEC), Interdisciplinary Nucleus for Physiology, Biophysics and Pathophysiology – Institute of Biomedical Science, Neuroscience and East Neuroscience Departments, Faculty of Medicine, University of Chile, Santiago, Chile; 29 Medicina interna sur, Facultad de Medicina, Universidad de Chile, Santiago, Chile; 30 Pritzker School of Medicine, The University of Chicago, Illinois, IL, United States; 31 Harvard T.H. Chan School of Public Health, Harvard University, Chicago, MA, United States; 32 Cognitive Neuroscience Center (CNC), Universidad de San Andrés, Buenos Aires, Argentina; 33 Department of Biophysics, School of Medicine, Istanbul Medipol University, Istanbul, Türkiye

**Keywords:** cognitive decline, cognitive outcomes, hormone therapy, Latin America and Caribbean, menopausal, reproductive history, systematic review, women’s brain health

## Abstract

Reproductive life stages, menopausal symptoms, and hormonal exposures are increasingly recognized as potential determinants of cognitive outcomes in women. However, these associations remain largely understudied in Latin American and Caribbean (LAC) populations. In this systematic review, we identified 18 observational studies published between 2003 and 2025. Of these, 16 were conducted across 13 LAC countries and two were conducted among latina women in the United States. Evidence was organized across four reproductive life stages domains: reproductive history, menopausal hormone therapy use, type of menopause and menopausal symptoms, and postmenopausal factors. Most LAC studies were cross-sectional and demonstrated substantial methodological heterogeneity. Across studies, shorter reproductive span, earlier menopause, higher parity, surgical menopause, and greater menopausal symptom burden were generally associated with poorer cognitive performance or higher odds of mild cognitive impairment. Some studies suggested favorable associations between menopausal hormone therapy use and cognitive outcomes. Postmenopausal factors, such as sarcopenia and obesity, as well as a longer duration of menopause, were predominantly linked to poorer cognitive outcomes. Only one population-based cohort evaluated incident dementia, underscoring the scarcity of longitudinal evidence in the region. Overall, the evidence suggests that reproductive life stages in LAC women are meaningfully associated with cognitive outcomes, reflecting the influence of hormonal transitions, menopausal characteristics, and postmenopausal health within distinctive regional contexts. However, the predominance of cross-sectional designs and limited regional representativity, coupled with a lack of integrated physiological indicators, reveal a substantial knowledge gap. Longitudinal and regionally representative cohorts incorporating standardized assessments of reproductive life stages alongside comprehensive cognitive measures are essential to understanding how reproductive life stages shape cognitive outcomes in LAC women.

**Systematic review registration**: The systematic review was registered in the Open Science Framework (OSF). Registration DOI/unique identifier: https://doi.org/10.17605/OSF.IO/ME7XK.

## 1 Introduction

Women represent approximately two-thirds of all individuals living with Alzheimer’s disease (AD) and have nearly twice the lifetime risk of developing the disease compared to men (Moutinho, 2025). In addition to higher incidence, women tend to experience faster disease progression, greater functional decline, and a substantially higher caregiving burden (Moutinho, 2025; Bourzac, 2025; Ferretti et al., 2018). These global patterns underscore pronounced sex differences in brain aging and dementia risk (Ribeiro et al., 2022). In Latin America and the Caribbean (LAC), a meta-analysis estimated the pooled prevalence of dementia among older adults at 10.6%. In the same study, crude prevalence estimates were 8.97% among women and 7.26% among men (Ribeiro et al., 2022). Within this regional context, understanding the determinants of cognitive disparities among women constitutes an urgent scientific and public health priority (Moutinho, 2025; Bourzac, 2025; Ferretti et al., 2018; Lutshumba et al., 2023).

Reproductive life stages have emerged as potential contributors to women’s cognitive vulnerability across adulthood and aging (Harlow et al., 2012; Soules et al., 2001). These stages encompass four major domains relevant to cognitive aging: reproductive history (e.g., age at menarche and menopause, parity, reproductive span, and hormonal contraceptive use), menopausal transition characteristics and symptom burden, menopausal hormone therapy (MHT) use, and postmenopausal health factors including cardiometabolic, muscular, and sleep-related conditions (Harlow et al., 2012; Inman and Flaws, 2024; Crockford et al., 2025). Together, these domains reflect cumulative endogenous and exogenous hormonal exposures across the life course and may influence cognitive outcomes through interconnected biological and clinical pathways (Mosconi et al., 2024; Raikes et al., 2025). Importantly, menopausal symptoms such as vasomotor disturbances, mood changes, and sleep alterations have been associated with neurocognitive changes and functional impairment (Crockford et al., 2025; Thurston et al., 2024), positioning the midlife reproductive transition as a potentially relevant window for brain health in later-life.

Emerging evidence, primarily from cohorts in North America and Europe, indicates that shorter reproductive span, earlier menopause, higher parity, and surgical menopause may be associated with poorer cognitive outcomes, while longer reproductive span and heterogeneity of hormonal exposure have been linked to more favorable cognitive trajectories (Moutinho, 2025; Bourzac, 2025; Barth et al., 2023). Mechanistic studies suggest that estrogen decline during menopause affects brain metabolic and neuroplastic processes relevant to cognitive function (Mosconi et al., 2024; Raikes et al., 2025; Manly et al., 2000). However, most of this evidence originates from populations in the Global North (Melville et al., 2025; Andy et al., 2024; Han et al., 2023), and its applicability to regions with distinct reproductive, demographic, and structural profiles remains uncertain.

This gap is particularly relevant in LAC, where reproductive trajectories and structural determinants differ substantially from those described in high-income settings. Women in the region have historically experienced higher completed fertility, earlier reproductive transitions, and earlier age at menopause (Islam et al., 2025; United Nations Department of Economic and Social Affairs, Population Division, 2020). At the same time, marked gender-based inequities in education, access to reproductive and menopausal care, and broader social determinants of health may modify the relationship between reproductive exposures and cognitive outcomes (Legaz et al., 2024; Gonzalez-Gomez et al., 2025; Baez et al., 2024). Despite these distinctive characteristics, few studies in LAC have systematically examined reproductive history, menopausal characteristics, MHT use, and postmenopausal health factors within a unified life-course framework. Existing studies are often cross-sectional, methodologically heterogeneous, and limited in regional representativeness, and no comprehensive synthesis has evaluated how these reproductive domains jointly relate to cognitive outcomes in women in LAC and Latina women outside LAC.

Therefore, this systematic review aims to synthesize and critically evaluate the available evidence on reproductive life stages, menopausal transition characteristics and symptoms, menopausal hormone therapy use, and postmenopausal health factors in relation to cognitive outcomes among women residing in LAC, and latin women living in the United States (hereafter LAC women). By organizing the evidence across these four predefined domains, this review seeks to clarify the scope, methodological features, and limitations of the current literature and to inform future research on LAC women’s brain health.

## 2 Methodology

This systematic review was conducted in accordance with the *Preferred Reporting Items for Systematic Reviews and Meta-Analyses* (PRISMA) guidelines (Page et al., 2021) (Figure 1) and was registered in the Open Science Framework (OSF) doi:10.17605/OSF. IO/ME7XK. The objective was to synthesize and critically evaluate the available evidence on reproductive life stages across the life course, including reproductive history, menopausal transition characteristics and symptoms, menopausal hormone therapy use, and postmenopausal factors and cognitive outcomes (cognitive performance, mild cognitive impairment, and dementia) among women residing in LAC, and latina women residing in the United States.

**Figure 1:**
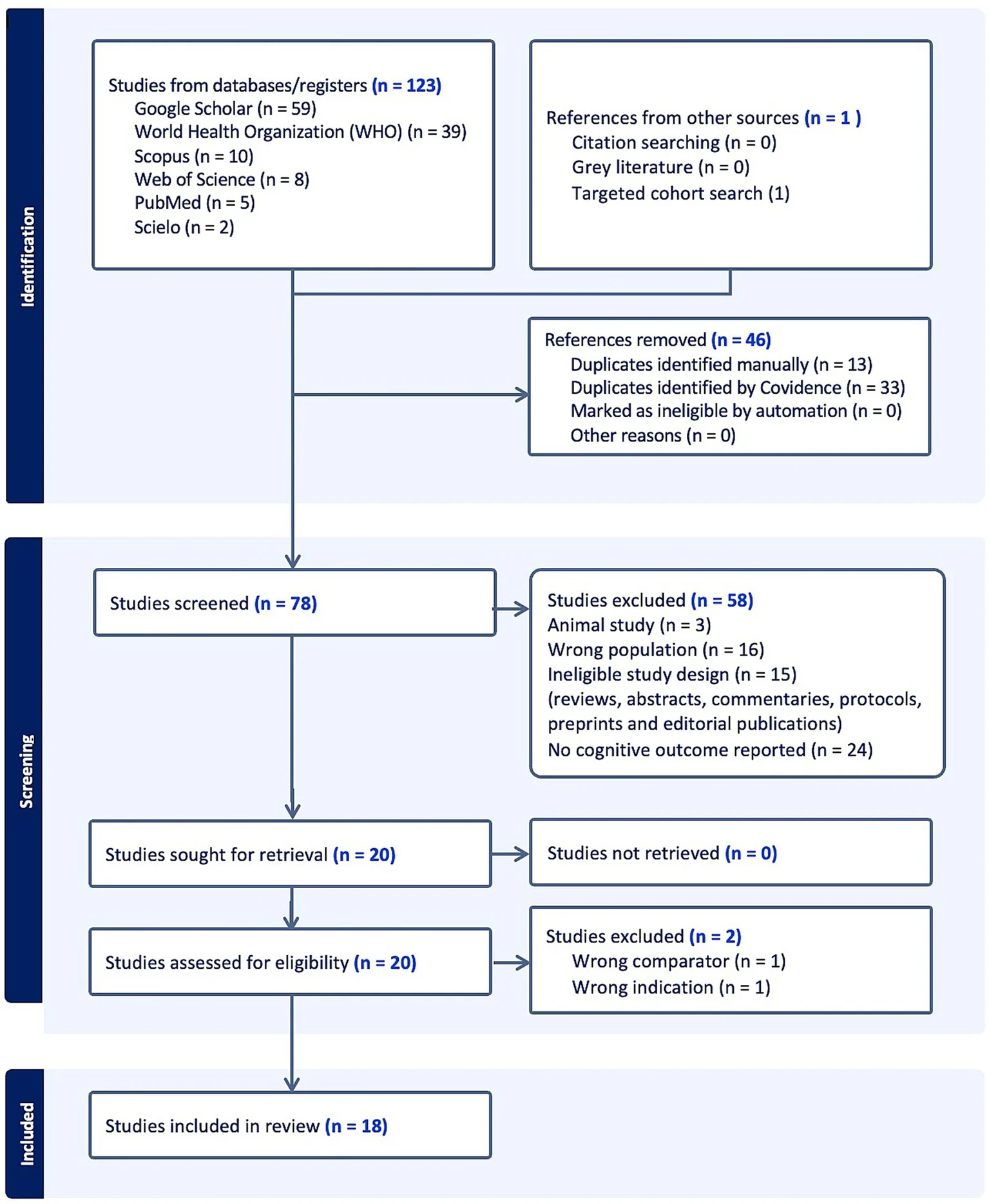
PRISMA (Page et al., 2021) flow diagram showing the process of identification, screening, eligibility, and inclusion of studies in the systematic review. References were identified through the following databases: Google Scholar, Redalyc, Scopus, Web of Science, PubMed, and Scielo. After removing duplicates, studies were screened and assessed for eligibility, resulting in the inclusion of 18 studies in the final review.

### 2.1 Search strategy

A comprehensive literature search was conducted in PubMed, Scopus, Web of Science, SciELO, Redalyc, and Google Scholar. The search covered records from database inception through November 17–28, 2025. An updated search was subsequently conducted on June 9–13, 2026. The final search strategy combined terms related to reproductive history and reproductive-life exposures, including menopause, menopausal hormone therapy, pregnancy complications, birth spacing, and contraceptive use, together with cognition and dementia-related terms, incorporating keywords in Spanish, English, and Portuguese (Supplementary Table S1). The search strategy was adapted to the structure of each database.

### 2.2 Study selection and deduplication

All records were imported into Covidence for deduplication and screening. Duplicates were removed using (Covidence, n.d.) automated process followed by manual verification when needed. Titles/abstracts were screened independently by two reviewers (NMD and CP), followed by full-text review of potentially eligible articles. Duplication and inclusion disagreements were resolved by consensus or consultation with a third reviewer (JC). Reasons for full-text exclusions were documented.

### 2.3 Eligibility criteria

We included observational studies (cross-sectional, case–control, and cohort designs, including longitudinal analyses) that met the following criteria: *Population*: Women residing in LAC, as well as studies composed exclusively of women of LAC origin residing in the United States, as defined by the original studies. *Exposure:* At least one exposure related to reproductive life stages across the life course (Harlow et al., 2012; Soules et al., 2001), including: (a) reproductive phase characteristics (e.g., age at menarche or menopause, reproductive span, parity, age at first/last birth, hormonal contraceptive use); (b) menopausal transition characteristics defined as factors related to the timing and experience of the menopausal transition (type of menopause and menopausal symptom burden, including vasomotor, mood, and sleep-related symptoms); (c) MHT use; and/or (d) postmenopausal, cardiometabolic, muscular, or sleep-related factors examined in relation to menopausal or reproductive status, defined as exposures assessed after the final menstrual period. Postmenopause was defined as the period following 12 consecutive months of amenorrhea not attributable to other causes. *Outcomes:* Cognitive outcomes defined within three domains: (i) cognitive performance measured through validated screening tools or neuropsychological batteries (Livingston et al., 2020); (ii) mild cognitive impairment (MCI), defined clinically or using validated thresholds (Jessen et al., 2014); and (iii) dementia diagnosis or incident dementia based on established clinical criteria (Jack et al., 2018). Subjective cognitive decline was included when operationalized using standardized criteria. We excluded reviews, editorials, case series, conference abstracts without full data, and studies lacking standardized cognitive outcomes.

#### 2.3.1 Data extraction and quality assessment

Two reviewers (NM and CP) independently screened titles/abstracts, reviewed full texts, and extracted data using a pre-piloted form within Covidence systematic review software (Veritas Health Innovation, Melbourne, Australia) (Covidence, n.d.). Extracted information included study design, country, sample size, population characteristics, exposure definitions, cognitive outcomes, and main findings. Discrepancies were resolved by consensus or consultation with a third reviewer (JC). The risk of bias was assessed using the Newcastle-Ottawa Scale (NOS), applying the standard NOS versions for cohort and observational studies, and an adapted NOS version for cross-sectional studies, as previously described in the literature (Wells et al., 2021).

### 2.4 Data synthesis

Given the methodological and clinical heterogeneity of the included studies, a standardized data extraction matrix was used to systematically collect descriptive and analytical characteristics of each study, including bibliographic information, study design, population characteristics, exposure definitions, cognitive outcomes, assessment instruments, covariates, and main findings, as reported by the original authors. The evidence was synthesized according to four predefined domains: reproductive history, menopausal hormone therapy use, type of menopause and menopausal symptoms, and postmenopausal factors related to metabolic, muscular, and sleep domains (Figure 2). Within each domain, findings were qualitatively compared across studies, with particular attention to the direction and consistency of reported associations, study design, exposure definitions, and operationalization of cognitive outcomes (Table 1).

**Figure 2:**
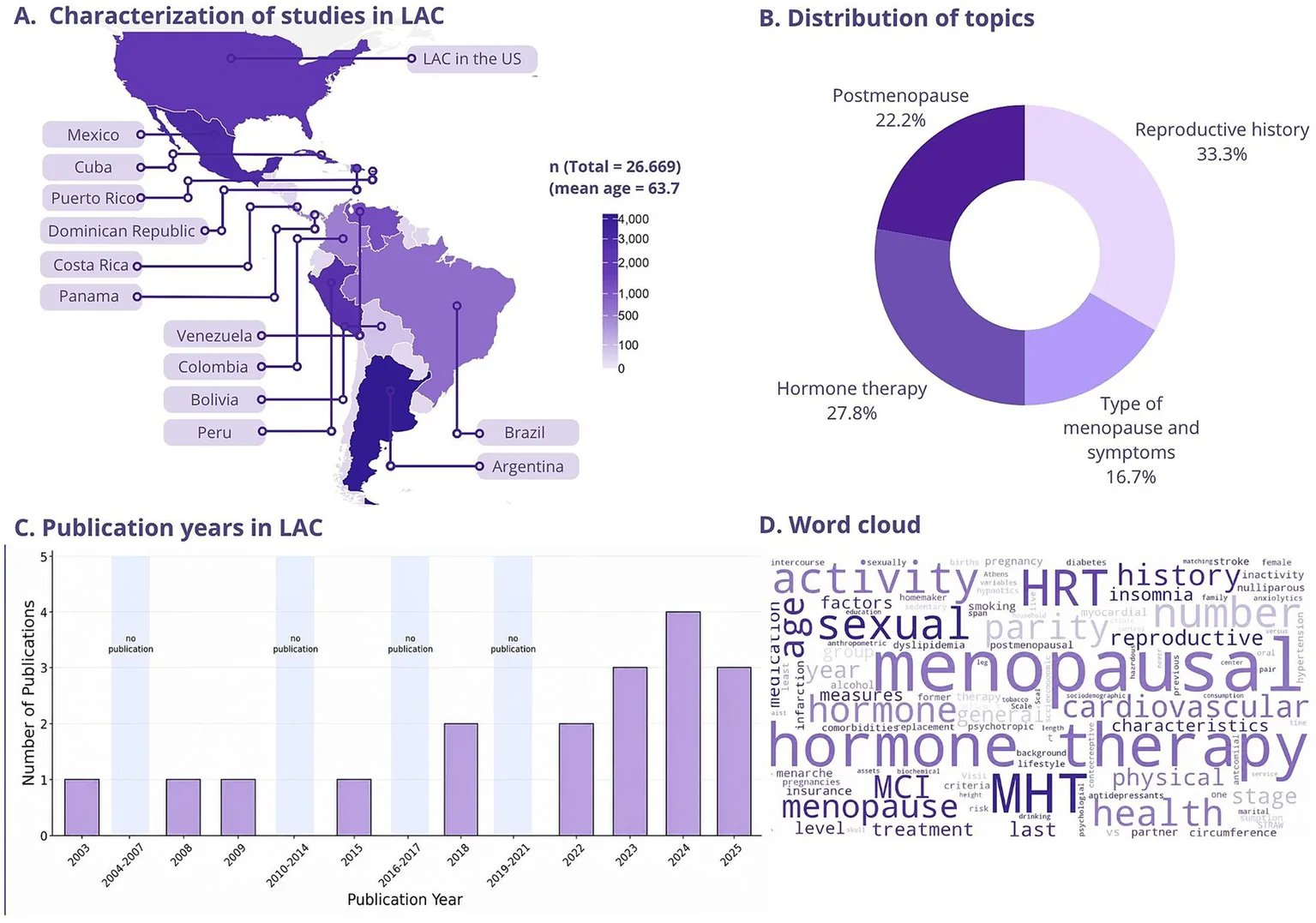
Overview of the studies included in this review. **(A)** Geographic distribution of included studies across Latin America and the Caribbean, with shading intensity representing the number of women included in each study location; the panel also summarizes the overall sample size, mean age, and mean years of education across studies. **(B)** Distribution of studies according to the reproductive exposure domain evaluated, including reproductive history, type of menopause and menopausal symptoms, menopausal hormone therapy, and postmenopausal factors. **(C)** Temporal distribution of included studies by publication year, illustrating trends in the available literature and periods in which no eligible studies were identified. **(D)** Bibliometric word cloud illustrating the frequency and co-occurrence of terms across the included literature. Word size reflects thematic frequency rather than the relative importance of specific exposures, outcomes, or associations.

**Table 1:** Summary of the empirical studies identified in this review examining the relationship between menopause-related factors and cognitive performance among Latin American and Latin women.

Study	Country	Design	n	Age	Education	Exposure	Outcome	Instruments	Finding
Monterrosa-Castro et al. (2025)	Argentina, Bolivia, Brazil, Colombia, Costa Rica, Ecuador, Mexico, Panama and Peru	Cross-sectional	1,185	56.9	13.6 ± 5.0	Sleep disturbances; postmenopause; menopausal symptoms; MCI; MHT; education; parity	MCI	MoCA	Severe sleep problems were independently associated with higher odds of mild cognitive impairment.
Blümel et al. (2025)	Argentina, Bolivia, Brazil, Colombia, Costa Rica, Ecuador, Mexico, Panama, and Peru	Cross-sectional	782	56.9	13.9	Hysterectomy without Oophorectomy and MHT use	Global cognition	MoCA	Bilateral oophorectomy was associated with higher odds of MCI.
Vallejo et al. (2025)	Argentina, Bolivia, Brazil, Colombia, Costa Rica, Ecuador, Mexico, Panamá, and Peru	Cross-sectional	1,185	56.9	13.6	Sarcopenia risk (SARC-*F* ≥ 4)	MCI	MoCA	Sarcopenia had a significantly association with mild cognitive impairment
Barbosa et al. (2024)	Brazil	Cross-sectional	2,594	69.58 ± 7.33	School attainment 4.58	Reproductive period length; parity; HRT use; age at menopause	Dementia status	ELSI-Brazil dementia algorithm (cognitive tests + IADLs/IQCODE)	High parity was associated with higher dementia odds, while MHT showed age-dependent associations: lower odds at 70–79 and higher odds at 80–89.
Blümel et al. (2025)	Argentina, Bolivia, Brazil, Colombia, Costa Rica, Ecuador, Mexico, Panama and Peru	Cross-sectional	722	56.9	13.6	Severe Obesity and Severe Menopausal Symptoms	MCI	MoCA	Severe obesity and severe menopausal symptoms increased the risk of cognitive impairment
Stickel et al. (2024)	Latin American women in the U. S.	Longitudinal	2,126	44–75	<High 40.1 High 19.0 >High 40.9	Menarche; pregnancies; births; oral contraceptive use; age at menopause; reproductive span and female hormone use	MCI	SEVLT, Word Fluency Test and DSST	A higher number of pregnancies and births was associated with worse processing speed
Calle et al. (2024)	Nine Latin American countries	Cross-sectional	1,287	55.5	13.8	Severe menopausal symptoms	MCI	MoCA	Severe menopausal symptoms were strongly associated with increased odds of mild cognitive impairment.
Espinoza et al. (2024)	Argentina, Bolivia, Brazil, Colombia, Costa Rica, Ecuador, Mexico, Panama, and Peru	Cross-sectional	1,185	55.3	13.3	Type of menopause; age at menopause; hysterectomy; oophorectomy; MHT use.	MCI	MoCA	Surgical menopause was associated with higher odds of mild cognitive impairment.
Universidad Nacional Mayor de San Marcos et al. (2023)	Peru	Cross-sectional	775	52.1 ± 5.7	Secondary education 35.6% higher and university education 48.9%	Menopausal status And menopausal symptoms.	Cognitive Impairment	MSSI	A greater severity of subjective cognitive symptoms during the menopausal transition, particularly in postmenopause
Cortés et al. (2023)	Latin American women in the U. S.	Longitudinal	3,621	59.2 ± 0.25	<High school 40.9 High school 19.6 More than high school 39.5	Age at menopause; reproductive period duration and age at menarche.	Global cognitive performance	SIS; B-SEVLT; Word Fluency Test and DSST	Age at menarche was inversely related to measures of executive function and processing speed.
Pérez Sampayo et al. (2022)	Mexico	Cross-sectional	394	49–59	Primary education: 41.9% Secondary education: 47.7% High school: 7.6% Bachelor’s degree: 2.5%	Insomnia	Cognitive impairment	MoCA	Insomnia was significantly associated with cognitive impairment
Blümel et al. (2022)	Argentina, Brazil, Bolivia, Mexico, Panama, and Peru	Cross-sectional	941	66.1 ± 5.8	12.4 ± 5.0	Age; parity; bilateral oophorectomy; MHT use; hypertension and sexual activity.	MCI	MoCA	Bilateral oophorectomy was significantly associated with mild cognitive impairment.
Prince et al. (2018)	Cuba, Dominican Republic, Puerto Rico, Peru, Mexico and Venezuela	Longitudinal	8,466	73.8	42,8% incomplete primary education.	Reproductive period; Parity and endogenous estrogen exposure	Incident dementia	CSI-D and CERAD Word List Learning	Greater gender parity was positively associated with the incidence of dementia.
Carranza-Lira and Carpio-Bárcenas (2018)	Mexico	Cross-sectional	94	60.93	By group: 56.9 ± 4.7 MHT; 66.8 ± 10.2 no MHT	Time since menopause; MHT use and age	Global cognition	MMSE	Longer time since menopause was associated with poorer cognitive performance.
Zilberman et al. (2015)	Argentina	Cross-sectional	1,034	47.13	Uneducated 17.4 Grammar school 48.1 Middle/high school 35.2 College 14.7	Hypertension; menopausal status; and MHT use	Cognitive Impairment and cortical function	MMSE; Benton Orientation Test; Clock-Drawing Test and Alternating Series Test	Hypertensive menopausal women performed worse on cognitive tests.
Fernandes et al. (2009)	Brazil	Cross-sectional	156	40–65	Schooling reported, no pooled mean verified	Menopausal status; arterial hypertension and schooling	Global cognition	MMSE and Word List Memory Test	Women in menopause showed lower scores in the domains of attention/calculation and immediate memory.
Pereira Pérez et al. (2008)	Colombia	Cross-sectional	50	50–55	Matched by education; no mean reported	MHT use	Short-term memory and visual and attention	TAVEC; BVRT; Digit Span Test And ROCF	Menopausal women who did not use MHT had worse cognitive performance
Hómez de Delgado et al. (2003)	Venezuela	Longitudinal	72	55–79	5.8	Menopausal hormone replacement therapy	Global cognitive function	MMSE, Benton, Boston.	One year of MHT did not improve cognitive function between groups.

The table presents the first author, publication year, study setting, design, and the main cognitive instruments used across studies.

## 3 Results

A total of 18 studies conducted among 26,314 women from LAC countries (*N* = 16; 89.5%; *n* women: 20,557) and Latina women residing in the United States were included (*N* = 2; 10.5%; n women: 5,757). These studies comprised single-country investigations from Argentina (Zilberman et al., 2015), Brazil (Fernandes et al., 2009; Barbosa et al., 2024), Colombia (Pereira Pérez et al., 2008), Mexico (Pérez Sampayo et al., 2022; Carranza-Lira and Carpio-Bárcenas, 2018), Peru (Universidad Nacional Mayor de San Marcos et al., 2023), and Venezuela (Hómez de Delgado et al., 2003), as well as large multicenter studies from the REDLINC X and REDLINC XII networks (Blümel et al., 2025), which pooled data from nine LAC countries. Evidence from the 10/66 Dementia Research Group cohort (Prince et al., 2018), including urban and rural sites in Mexico and Peru and urban sites in Puerto Rico and Venezuela, was also incorporated. In addition, studies of Latino populations residing in the United States participating in the Hispanic Community Health Study/Study of Latinos (HCHS/SOL) and SOL-INCA were included (Cortés et al., 2023; Stickel et al., 2024) from Dominican Republic, Cuba, Mexico, Puerto Rico and Venezuela (Figure 2).

### 3.1 Quality assessment

A quality assessment using the Newcastle–Ottawa Scale (NOS) (Blümel et al., 2025; Zanini et al., 2014) showed that most studies had satisfactory methodological quality, with 69% (11/16) scoring 5–6 stars, while only a few achieved a rating of good quality (≥7 stars; 31% [5/16]) (Supplementary Table S2).

Across NOS domains, scores were consistently higher for selection and exposure or outcome ascertainment, whereas the comparability domain showed systematically lower ratings, with most studies receiving one to two stars (Supplementary Table S2).

### 3.2 Reproductive history

Reproductive history is conceptualized as the set of reproductive events and characteristics across a woman’s lifespan, including age at menarche, reproductive lifespan, parity, age at childbirth, menopause-related factors, and hormonal exposures, which together reflect cumulative hormonal exposure and have been linked to cardiometabolic, musculoskeletal, and cognitive risks (Vallejo et al., 2025; Prince et al., 2018; Cortés et al., 2023). This conceptualization, grounded in the *Stages of Reproductive Aging Workshop* (STRAW) criteria (Harlow et al., 2012; Soules et al., 2001), aligns with contemporary epidemiological evidence linking these indicators with cognitive health across the life course (Han et al., 2023; Stickel et al., 2024).

Regarding parity, evidence from the 10/66 Dementia Research Group study (Prince et al., 2018), which included 8,466 women aged 65 years and older, showed that the risk of dementia increased linearly with each additional live-born child (adjusted subhazard ratio [ASHR] = 1.03; 95% CI: 1.02–1.06), suggesting that high parity may contribute to cognitive vulnerability in late life. A similar result was found in the REDLINC XII cohort (Flascym Federación Latinoamericana Climaterio Menopausia, n.d.), where postmenopausal women with more children were more likely to show MCI (means 2.2 ± SD 1.7 vs. 2.6 ± SD 1.7 children) than those with fewer children (Espinoza et al., 2024). In Brazil, a cross-sectional analysis of 2,594 women aged ≥60 years from the ELSI-Brazil cohort found that women with 5–8 childbirths had higher odds of dementia compared to nulliparous women (OR = 2.55; 95% CI: 1.09–5.98), while reproductive period length and continuous parity were not significantly associated with dementia status (Barbosa et al., 2024). The number of living children was also independently associated with higher dementia odds (OR = 1.08; 95% CI: 1.01–1.16). Taken together, these findings position parity as a central reproductive exposure in the region’s cognitive aging landscape. The SOL-INCA study (Graves et al., 2024) conducted in 2,126 Latina women in the United States over 7 years, expanded upon these findings by analyzing multiple reproductive exposures (Stickel et al., 2024). The results showed that a higher number of pregnancies and live births were associated with poorer performance on processing speed tests (Pregnancies: *β* = −0.023, SE = 0.011, *p* < 0.05; Live births: *β* = −0.044, SE = 0.014, *p* < 0.01), a cognitive domain particularly vulnerable to aging and neurodegenerative processes. Conversely, the use of oral hormonal contraceptives was associated with better global cognitive performance [*β* Global = 0.124 (SE = 0.040), *p* < 0.01], independent of the number of births (Stickel et al., 2024). Data from Latina populations living in the United States complemented these results (Fu et al., 2022). The HCHS/SOL (Study of Latinos, n.d.), which assessed 3,630 women aged 45–74 years, also provided evidence for the relationship between reproductive span and cognitive function (Cortés et al., 2023). In this diverse population, a later age at menopause and a longer reproductive span were associated with better performance on tests of verbal memory, learning (𝛽 = 0.04; SE = 0.02; *p* < 0.05), and processing speed (𝛽 = 0.10; SE = 0.05; *p* < 0.0001). This pattern persisted after adjusting for socioeconomic and lifestyle factors, underscoring the potential neuroprotective role of prolonged endogenous estrogen exposure (Cortés et al., 2023; Stickel et al., 2024) (Figure 3).

**Figure 3:**
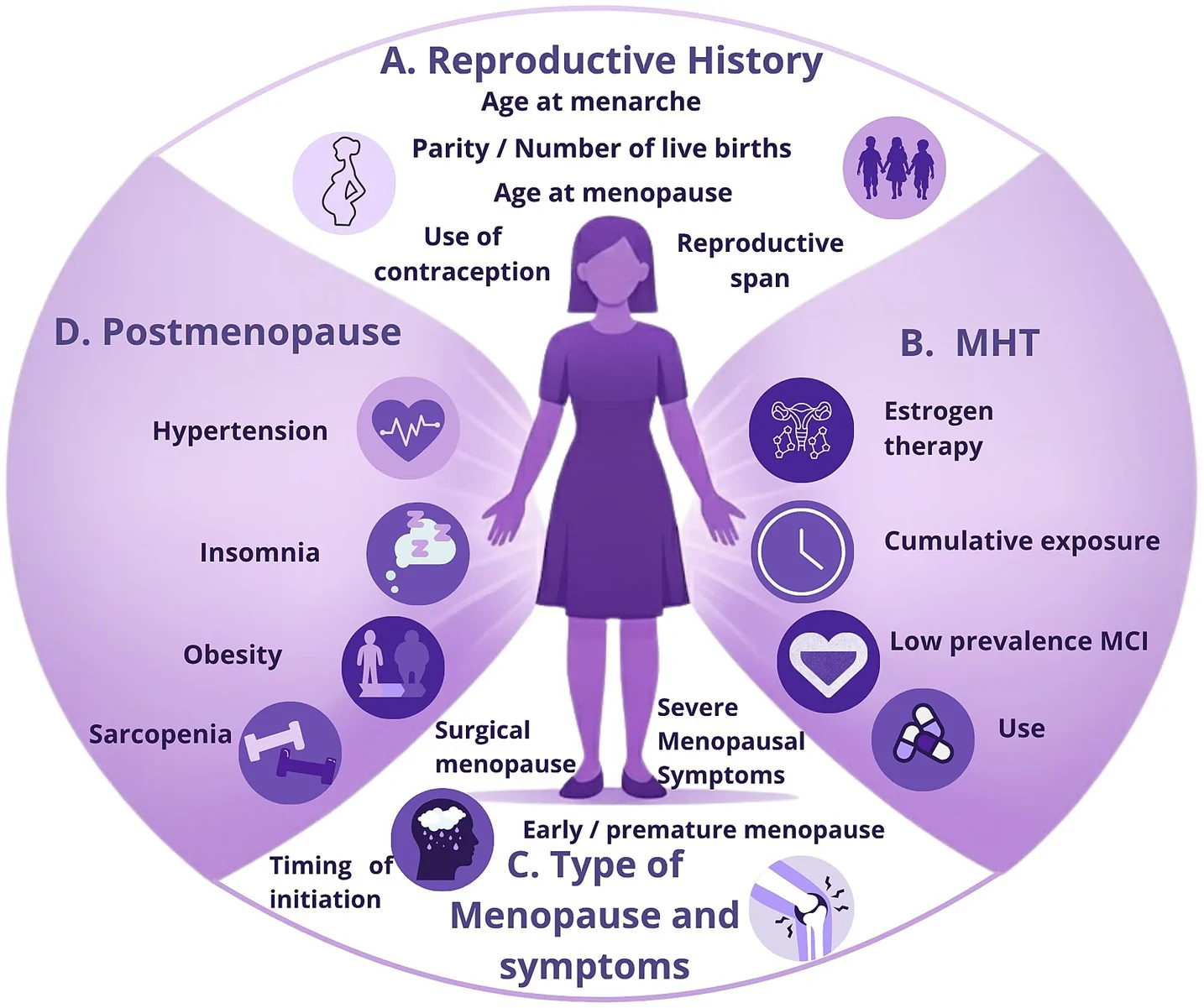
Conceptual synthesis of the reproductive life-stage domains identified in this review and their associations with cognitive outcomes. **(A)** Reproductive history: Impact of age at menarche and menopause, parity, contraceptive use, and reproductive span. **(B)** MHT: Influence of estrogen type, cumulative exposure, and the timing of initiation on the risk of MCI. **(C)** Type of menopause and symptoms: Relevance of early, premature, or surgical menopause, and the burden of severe menopausal symptoms as modifiers of cognitive outcomes. **(D)** Postmenopausal factors: Interrelation between hypertension, obesity, sarcopenia, and insomnia, and their associated metabolic pathways influencing cognitive reserve.

### 3.3 Menopausal hormone therapy

MHT refers to the administration of estrogens, with or without progestogens, delivered systemically or locally to relieve menopausal symptoms or prevent chronic conditions associated with estrogen deficiency (Mukherjee and Davis, 2025; Panay et al., 2024). Key variables include the type of formulation (estrogen-only, combined estrogen-progestogen, or tibolone), route of administration (oral, transdermal, or vaginal), age at initiation, timing relative to menopause onset, and total duration of treatment (Melville et al., 2025; Nerattini et al., 2023). This definition aligns with the recommendations of international clinical guidelines and large-scale epidemiological evidence linking MHT to cognitive outcomes (Barth et al., 2023; Mills et al., 2023; Rocca et al., 2008; Manson et al., 2017).

One multicenter study (Flascym Federación Latinoamericana Climaterio Menopausia, n.d.), which included 941 postmenopausal women aged ≥60 years, evaluated the association between MHT use and MCI, defined using the Montreal Cognitive Assessment (MoCA). Among women with intact ovaries, 29.3% of MHT non-users presented with MCI compared to 11.7% of MHT users. Among those with bilateral oophorectomy, the difference was even greater: 45.2% vs. 12.8% for non-users vs. users. After adjusting for age, education, parity, and vascular risk factors, MHT use remained independently associated with lower prevalence of MCI (OR = 0.18; 95% CI: 0.10–0.32) alongside ≥12 years of education (OR = 0.46; 95% CI: 0.32–0.65) and regular sexual activity (OR = 0.56; 95% CI: 0.40–0.79) (Blümel et al., 2022). These findings were supported by two additional studies (Calle et al., 2024; Flascym Federación Latinoamericana Climaterio Menopausia, n.d.). In Colombia, a quasi-experimental study in 50 menopausal women aged 50–55 years compared users and non-users of estrogen therapy (Pereira Pérez et al., 2008). Women receiving MHT performed better on neuropsychological tests assessing short-term memory (mean score 6.00 vs. 4.72; *p* < 0.001), visual attention (mean score 29.40 vs. 27.12; *p* = 0.016), and auditory attention (mean score 70.40 vs. 65.68; *p* < 0.001), suggesting selective cognitive benefits in domains sensitive to estrogenic action. Similarly, analysis of the SOL-INCA cohort showed that the use of oral MHT was associated with a lower prevalence of MCI (OR = 0.35; 95% CI: 0.13–0.94), reinforcing the potential neuroprotective role of exogenous hormonal exposure throughout life (Stickel et al., 2024) (Figure 3).

### 3.4 Type of menopause and menopausal symptoms

Type of menopause was defined according to clinical criteria distinguishing natural menopause, surgical menopause (bilateral oophorectomy with or without hysterectomy), and early or premature menopause. Menopausal symptoms were understood as the cluster of vasomotor, psychological, somatic, sexual, and sleep-related symptoms arising from estrogen depletion, with recognized implications for cardiovascular, metabolic, and cognitive health (Harlow et al., 2012; Soules et al., 2001; NICE, 2019). Early menopause is considered to occur before the age of 45 years (Than et al., 2023; Conde et al., 2021), while surgical menopause is defined as that induced by bilateral oophorectomy with or without hysterectomy (Giannakaki et al., 2025; Shao et al., 1999). These characteristics have been linked to an increased risk of cognitive decline due to accelerated loss of endogenous estrogens and the interplay with vascular and metabolic factors (Crockford et al., 2025; Auro et al., 2014).

The most representative study was a multicenter analysis (Flascym Federación Latinoamericana Climaterio Menopausia, n.d.) of 1,287 women from nine LAC countries, which used the MoCA to evaluate MCI (Calle et al., 2024). This study found that women with severe menopausal symptoms (measured using the Menopause Rating Scale) had twice the risk (OR = 1.74; 95% CI: 1.25–2.42) of developing MCI compared with those with mild or moderate symptoms. Factors such as higher educational level (OR = 0.31; 95% CI: 0.21–0.46), regular physical exercise (OR = 0.55; 95% CI: 0.39–0.76) and sexual activity (OR = 0.70; 95% CI: 0.51–0.96), and lower body mass index (BMI) were associated with a significant reduction in the risk of MCI (OR = 0.96; 95% CI: 0.95–0.98), highlighting the complex interplay between menopausal symptoms and social and clinical determinants of cognitive health (Calle et al., 2024). Additionally, an analysis of 1,185 women from the same cohort (Flascym Federación Latinoamericana Climaterio Menopausia, n.d.) showed that surgical menopause, regardless of age at surgery, was associated with lower cognitive scores and a higher risk of MCI (OR = 1.47; 95% CI: 1.01–2.16) compared with spontaneous menopause (Espinoza et al., 2024). Within the same cohort (Flascym Federación Latinoamericana Climaterio Menopausia, n.d.), a subanalysis of 772 women revealed that sarcopenia (measured using the SARC-F questionnaire) and severe menopausal symptoms were associated with markedly higher MCI prevalence (35.3%) (OR = 2.44; 95% CI: 1.50–3.95) compared to women without these conditions (12.9%) (Vallejo et al., 2025).

A multicountry sub-analysis, including 782 LAC women (Flascym Federación Latinoamericana Climaterio Menopausia, n.d.) (Blümel et al., 2025; Blümel et al., 2025) (Figure 3).

### 3.5 Postmenopause

Postmenopause begins after the final menstrual period and is characterized by persistently low estradiol levels. According to the Stages of Reproductive Aging Workshop criteria (Harlow et al., 2012; Soules et al., 2001), this stage represents a prolonged period of physiological adaptation marked by hormonal, metabolic, and cardiovascular changes that may impact cognitive health (Ryczkowska et al., 2022; Mehta and Manson, 2024; Auro et al., 2014). During this stage, factors such as years since menopause, the severity of persistent symptoms (e.g., vasomotor, genitourinary, and sleep disturbances), and the presence of common comorbidities such as obesity and sarcopenia may modify the risk of cognitive decline and dementia (Crockford et al., 2025; Nerattini et al., 2023; Than et al., 2023).

In Argentina, among 1,034 postmenopausal women, arterial hypertension was associated with a 48% higher risk of cortical dysfunction (OR = 1.48; 95% CI: 1.06–2.07) as measured by the Boston Naming Test (Zilberman et al., 2015). In Mexico, a study of 94 postmenopausal women found that those with a duration of more than 20 years since menopause onset, even adjusted for age, had significantly lower Mini-Mental State Examination (MMSE) scores compared to peers with shorter time since menopause (*r* = −0.873; *p* < 0.001), especially among those not using MHT (Carranza-Lira and Carpio-Bárcenas, 2018). Finally, a study of 39 postmenopausal women found that insomnia, a highly prevalent condition during this life stage, was associated with a threefold increase in the likelihood of cognitive decline (OR = 3.09; 95% CI: 1.47–6.50) (Pérez Sampayo et al., 2022). These findings reinforce the growing recognition that persistent sleep disturbances may have a profound impact on late-life brain health beyond affecting quality of life (Pérez Sampayo et al., 2022). A larger multinational sub-analysis of 1,185 postmenopausal women from nine LAC countries further corroborated these findings. In this study, severe sleep problems were assessed using either the sleep item of the Menopause Rating Scale (MRS) or the Jenkins Sleep Scale. Severe sleep problems were independently associated with higher odds of MCI in both adjusted models, whether assessed with the MRS sleep item (aOR = 1.81; 95% CI: 1.26–2.60) or the JSS (aOR = 1.88; 95% CI: 1.31–2.69). Physical inactivity and higher parity also emerged as independent risk factors, whereas ever-use of MHT (aOR = 0.37–0.38) and university education (aOR = 0.34) were independently associated with lower odds of MCI across both models (Monterrosa-Castro et al., 2025) (Figure 3).

## 4 Discussion

This PRISMA-guided systematic review provides a structured synthesis of reproductive life stages, menopausal symptoms, postmenopausal factors, and cognitive outcomes among women from LAC, thereby establishing the current regional status quo. Across 18 studies published between 2003 and 2025, earlier age at menopause and menarche, as well as higher parity, were more frequently associated with poorer cognitive performance or greater likelihood of mild cognitive impairment (Fernandes et al., 2009; Bazyar et al., 2025). Conversely, longer reproductive span and later age at menopause were generally linked to more favorable cognitive performance. Greater menopausal symptom burden, surgical menopause, and several postmenopausal cardiometabolic factors were also associated with worse cognitive outcomes (Fu et al., 2022; Study of Latinos, n.d.; Nerattini et al., 2023).

When interpreted alongside global literature, findings from LAC broadly align with prior studies reporting that shorter reproductive span and earlier menopause are associated with poorer cognitive performance and higher risk of mild cognitive impairment (Han et al., 2023; Ribeiro et al., 2023; Nabhan et al., 2022). Regarding MHT, several regional studies suggested favorable associations with cognitive performance or reduced likelihood of cognitive impairment, although a recent systematic review and meta-analysis did not demonstrate a clear protective or harmful effect of MHT on dementia risk (Melville et al., 2025). This divergence likely reflects heterogeneity in hormone formulations, timing of initiation, duration, route of administration, and potential healthy-user bias (Andy et al., 2024; Espeland et al., 2013; Gleason et al., 2024; Henderson et al., 2016; Kantarci et al., 2018). In LAC, this bias may be particularly pronounced, as access to MHT is often restricted to women with higher educational attainment and socioeconomic status, both of which independently contribute to greater cognitive reserve.

Although the overall direction of associations was generally consistent across studies, some variability was observed across reproductive domains. Associations involving reproductive lifespan and age at menopause tended to show more consistent relationships with cognitive outcomes and dementia risk (Prince et al., 2018; Cortés et al., 2023; Stickel et al., 2024), whereas findings regarding menopausal symptoms and MHT were more heterogeneous (Pereira Pérez et al., 2008; Hómez de Delgado et al., 2003; Espinoza et al., 2024; Blümel et al., 2022; Calle et al., 2024; Stickel et al., 2024). This variability may partly reflect differences in participant characteristics, including age, educational attainment, menopausal status, and underlying health conditions, as well as variation in the definition and measurement of reproductive exposures and cognitive outcomes. Additionally, disparities in healthcare access, socioeconomic conditions, and broader structural determinants across LAC settings may influence both reproductive experiences and cognitive aging trajectories (Legaz et al., 2024; Baez et al., 2024; World Population Review, 2025; Bazyar et al., 2025; América Latina, 1996; OECD, n.d.). Taken together, these findings suggest that reproductive influences on cognitive health are shaped by an interplay of biological and contextual factors, reinforcing the importance of a life-course perspective when interpreting evidence from LAC populations.

These findings must also be interpreted within the distinctive reproductive and sociobiological context of LAC (Ribeiro et al., 2023). Population-based data indicate that the mean age at menarche in LAC women is approximately 12.6 years, compared with 13.4 years reported in Europe (World Population Review, 2025; Bazyar et al., 2025). Women currently in mid and late life in LAC historically experienced higher completed fertility, commonly three to five live births per woman (América Latina, 1996), compared with 1.5 to 2.0 in OECD countries (OECD, n.d.). Natural menopause also occurs earlier in LAC, at approximately 48 to 49 years (Blümel et al., 2006), compared with 50 to 51 years in Europe and the United States (Palacios et al., 2010). This convergence of earlier menarche, greater cumulative parity, and earlier menopause defines a distinct reproductive window and cumulative hormonal trajectory. While the direction of the associations coincides with findings from global settings (Palacios et al., 2010), equivalence in magnitude or interaction cannot be assumed when considering the structural determinants specific to LAC. Research in LAC emphasizes that cognitive vulnerability stems from the intersection of reproductive history and structural inequality. Factors like low education and socioeconomic status may exert independent and substantial contributions to cognitive vulnerability, interacting with reproductive exposures across the life course rather than functioning as simple confounders, suggesting that reproductive aging in the LAC region (Santamaria-Garcia et al., 2023; Americas TLRH, 2023).

From a clinical and public health perspective in LAC, reproductive and menopausal characteristics may represent accessible indicators of cognitive vulnerability during midlife. Greater menopausal symptom burden, particularly vasomotor, sleep, and mood disturbances, may signal increased neurocognitive risk and support closer monitoring (Kazemi et al., 2022; Hazelton et al., 2025; Nguyen-Rodriguez et al., 2024; Quesada et al., 2024). Associations between reproductive history and cardiometabolic risk further highlight the importance of integrating reproductive health into broader cardiovascular and brain health prevention strategies. However, current evidence does not support specific recommendations regarding MHT for prevention of cognitive decline, emphasizing the need for individualized and timing-sensitive clinical decision-making (Ryczkowska et al., 2022; Mehta and Manson, 2024; Golombek et al., 2023).

This review also identifies important gaps and opportunities in the evidence base. Longitudinal studies evaluating incident dementia remain scarce, and characterization of hormonal exposures, including contraceptive use and MHT formulation, timing, and duration, was frequently incomplete (Pereira Pérez et al., 2008; Hómez de Delgado et al., 2003). Notably, no included studies specifically evaluated perimenopause as a distinct reproductive stage or reported cognitive outcomes separately for perimenopausal women, limiting current understanding of cognitive changes occurring during the menopausal transition. Female-specific reproductive conditions and cumulative environmental and social exposures remain underexplored, despite growing evidence linking these factors to biological aging and cognitive vulnerability (Fu et al., 2022; Ribeiro et al., 2023; Gregory et al., 2023; Pszczołowska et al., 2024; Gregory et al., 2025). Furthermore, the absence of neuroimaging, genetic, hormonal, and inflammatory biomarkers limits mechanistic interpretation and prevents direct evaluation of biological pathways. Several limitations of the available evidence must be acknowledged. Substantial methodological heterogeneity across studies, including variability in cognitive outcome definitions, exposure characterization, and covariate adjustment, precluded quantitative meta-analysis and limited comparability. The predominance of cross-sectional designs restricts causal inference and temporal interpretation. Additionally, a significant proportion of studies originated from specific research networks, and some cohorts included populations residing outside the LAC region, potentially limiting generalizability across diverse regional populations and healthcare contexts (Prince et al., 2018; Stickel et al., 2024). These factors underscore the need for standardized, longitudinal, regionally representative research.

Future research should prioritize longitudinal, biomarker-integrated cohort studies capable of evaluating causal pathways linking reproductive aging and cognitive outcomes. Integration of reproductive history with neuroimaging, hormonal, genetic, metabolic, inflammatory, and exposome-informed measures will be essential to clarify mechanisms. Existing regional initiatives, including (LAC-CD, 2021), LATAM-FINGERS (Crivelli et al., 2023), ReDLat (Ibanez et al., 2021), REDLINC (Flascym Federación Latinoamericana Climaterio Menopausia, n.d.), and Wellcome Leap CARE (CARE Program Details, n.d.) in LAC, provide an emerging infrastructure to support coordinated multicenter research and biomarker integration. Leveraging these platforms will be critical to generating globally relevant yet context-sensitive evidence.

In conclusion, reproductive aging in women from LAC should be understood as a fundamentally biosocial process shaped by cumulative hormonal exposure and structural vulnerability across the life course. Reproductive history and menopausal characteristics function as proxies for broader biological and social processes influencing brain health. However, the current evidence base remains limited by methodological heterogeneity and insufficient longitudinal data. Advancing the field will require integrative, biomarker-informed, life-course approaches capable of elucidating how reproductive trajectories and structural determinants jointly shape cognitive aging and resilience in women across the region.

## Data Availability

The original contributions presented in the study are included in the article/Supplementary material, further inquiries can be directed to the corresponding author.
