# Supplementary material for: Reproductive life stages and cognitive outcomes: a systematic review in Latin American and Caribbean women
Source: Front Aging Neurosci. 2026 Sep 17;18:1826530. doi: 10.3389/fnagi.2026.1826530 (PMC13627426; doi:10.3389/fnagi.2026.1826530)
Supplement: Supplementary file 1 [file Data_Sheet_1.docx]

***Supplementary Material***

## 1.1 Supplementary Tables

**Supplementary Table 1**: *Search Strategy and terms.* Keywords and search strategies used in the systematic review across multiple databases, the table details the specific search terms related to reproductive state and geographic regions, as well as how these terms were applied to each database.

| Database | Search-terms |
| --- | --- |
| PubMed | ("reproductive history"[Title/Abstract] OR "reproductive lifespan"[Title/Abstract] OR "reproductive span"[Title/Abstract] OR menopause[Title/Abstract] OR postmenopause[Title/Abstract] OR "surgical menopause"[Title/Abstract] OR "pregnancy complication*"[Title/Abstract] OR preeclampsia[Title/Abstract] OR "gestational diabetes"[Title/Abstract] OR "birth interval"[Title/Abstract] OR "interpregnancy interval"[Title/Abstract] OR contraception[Title/Abstract] OR contraceptive*[Title/Abstract] OR "oral contraceptive*"[Title/Abstract] OR "hormone therapy"[Title/Abstract] OR "hormone replacement therapy"[Title/Abstract] OR "estrogen therapy"[Title/Abstract]) AND (cognition[Title/Abstract] OR "cognitive decline"[Title/Abstract] OR "cognitive impairment"[Title/Abstract] OR dementia[Title/Abstract] OR "Alzheimer disease"[Title/Abstract] OR "mild cognitive impairment"[Title/Abstract]) AND ("Latin America"[Mesh] OR "South America"[Mesh] OR "Central America"[Mesh] OR "Argentina"[Mesh] OR "Bolivia"[Mesh] OR "Brazil"[Mesh] OR "Chile"[Mesh] OR "Colombia"[Mesh] OR "Costa Rica"[Mesh] OR "Cuba"[Mesh] OR "Dominican Republic"[Mesh] OR "Ecuador"[Mesh] OR "Guatemala"[Mesh] OR "Honduras"[Mesh] OR "Mexico"[Mesh] OR "Nicaragua"[Mesh] OR "Panama"[Mesh] OR "Paraguay"[Mesh] OR "Peru"[Mesh] OR "Puerto Rico"[Mesh] OR "Uruguay"[Mesh] OR "Venezuela"[Mesh]) |
| Scopus | TITLE-ABS-KEY ("reproductive history" OR "reproductive lifespan" OR "reproductive span" OR menopause OR postmenopause OR "surgical menopause" OR "pregnancy complication*" OR preeclampsia OR "gestational diabetes" OR "birth interval" OR "interpregnancy interval" OR contraception OR contraceptive* OR "oral contraceptive*" OR "hormone therapy" OR "hormone replacement therapy" OR "estrogen therapy") AND TITLE-ABS-KEY (cognition OR "cognitive decline" OR "cognitive impairment" OR dementia OR "Alzheimer's disease" OR "mild cognitive impairment") AND TITLE-ABS-KEY ("Latin America" OR "South America" OR "Central America" OR Argentina OR Bolivia OR Brazil OR Chile OR Colombia OR Costa Rica OR Cuba OR Ecuador OR Guatemala OR Honduras OR Mexico OR Nicaragua OR Panama OR Paraguay OR Peru OR Puerto Rico OR Uruguay OR Venezuela) |
| Web of science | TS=("reproductive history" OR "reproductive lifespan" OR "reproductive span" OR "historia reproductiva" OR "história reprodutiva" OR menopause OR menopausia OR menopausa OR postmenopause OR postmenopausia OR pós-menopausa OR "surgical menopause" OR "menopausia quirúrgica" OR "menopausa cirúrgica" OR "pregnancy complication*" OR "complicaciones del embarazo" OR "complicações da gravidez" OR preeclampsia OR preeclampsia OR pré-eclâmpsia OR "gestational diabetes" OR "diabetes gestacional" OR "birth interval" OR "intervalo entre nacimientos" OR "intervalo entre nascimentos" OR "interpregnancy interval" OR "intervalo intergenésico" OR "intervalo intergestacional" OR contraception OR anticoncepción OR contracepção OR contraceptive* OR anticonceptivo* OR contraceptivo* OR "oral contraceptive*" OR "anticonceptivos orales" OR "contraceptivos orais" OR "hormone therapy" OR "terapia hormonal" OR "terapia hormonal" OR "hormone replacement therapy" OR "terapia de reemplazo hormonal" OR "terapia de reposição hormonal" OR "estrogen therapy" OR "terapia con estrógenos" OR "terapia com estrogênios") AND TS=(cognition OR cognición OR cognição OR "cognitive decline" OR "deterioro cognitivo" OR "declínio cognitivo" OR "cognitive impairment" OR "deterioro cognitivo" OR "comprometimento cognitivo" OR dementia OR demencia OR demência OR "Alzheimer disease" OR "enfermedad de Alzheimer" OR "doença de Alzheimer" OR "mild cognitive impairment" OR "deterioro cognitivo leve" OR "comprometimento cognitivo leve") AND TS=("Latin America" OR "América Latina" OR Argentina OR Bolivia OR Brazil OR Brasil OR Chile OR Colombia OR "Costa Rica" OR Cuba OR Ecuador OR Guatemala OR Honduras OR Mexico OR México OR Nicaragua OR Panama OR Panamá OR Paraguay OR Peru OR Perú OR "Puerto Rico" OR Uruguay OR Venezuela) |
| SciELO | ("reproductive history" OR "reproductive lifespan" OR "reproductive span" OR "historia reproductiva" OR "história reprodutiva" OR "pregnancy complication*" OR "complicaciones del embarazo" OR "complicações da gravidez" OR preeclampsia OR pré-eclâmpsia OR "gestational diabetes" OR "diabetes gestacional" OR "birth interval" OR "intervalo entre nacimientos" OR "intervalo entre nascimentos" OR "interpregnancy interval" OR "intervalo intergenésico" OR "intervalo intergestacional" OR contraception OR anticoncepción OR contracepção OR contraceptive* OR anticonceptivo* OR contraceptivo* OR "oral contraceptive*" OR "anticonceptivos orales" OR "contraceptivos orais") AND (cognition OR cognición OR cognição OR "cognitive decline" OR "deterioro cognitivo" OR "declínio cognitivo" OR "cognitive impairment" OR "comprometimiento cognitivo" OR "comprometimento cognitivo" OR dementia OR demencia OR demência OR "Alzheimer disease" OR "enfermedad de Alzheimer" OR "doença de Alzheimer" OR "mild cognitive impairment" OR "deterioro cognitivo leve" OR "comprometimento cognitivo leve") AND ("Latin America" OR "América Latina" OR Argentina OR Bolivia OR Brazil OR Brasil OR Chile OR Colombia OR "Costa Rica" OR Cuba OR Ecuador OR Guatemala OR Honduras OR Mexico OR México OR Nicaragua OR Panama OR Panamá OR Paraguay OR Peru OR Perú OR "Puerto Rico" OR Uruguay OR Venezuela) |
| Redalyc | ("historia reproductiva" OR "história reprodutiva" OR "vida reproductiva" OR "vida reprodutiva" OR paridad OR "complicaciones del embarazo" OR "complicações da gravidez" OR preeclampsia OR "pré-eclâmpsia" OR "diabetes gestacional" OR "intervalo entre nacimientos" OR "intervalo entre nascimentos" OR "intervalo intergenésico" OR "intervalo intergestacional" OR anticoncepción OR contracepção OR anticonceptivo* OR contraceptivo* OR "anticonceptivos orales" OR "contraceptivos orais") AND ("deterioro cognitivo" OR "declínio cognitivo" OR demencia) AND ("América Latina" OR Latinoamérica OR "Latin America")  ("terapia hormonal" OR "terapia de reemplazo hormonal" OR "terapia de reposición hormonal") AND ("deterioro cognitivo" OR "declínio cognitivo" OR demencia) AND ("América Latina" OR Latinoamérica OR "Latin America")  ("menopausia" OR "menopausa") AND ("deterioro cognitivo" OR "declínio cognitivo" OR demencia) AND ("América Latina" OR Latinoamérica OR "Latin America")  ("postmenopausia" OR "posmenopausia" OR "pós-menopausa") AND ("deterioro cognitivo" OR "declínio cognitivo" OR demencia) AND ("América Latina" OR Latinoamérica OR "Latin America") |
| Google  scholar | ("reproductive history" OR "historia reproductiva" OR "história reprodutiva" OR "reproductive lifespan" OR "vida reproductiva" OR "vida reprodutiva" OR paridad OR parity OR menopause OR menopausia OR menopausa OR postmenopause OR postmenopausia OR "pós-menopausa" OR perimenopause OR perimenopausia OR perimenopausa OR "pregnancy complication*" OR "complicaciones del embarazo" OR "complicações da gravidez" OR preeclampsia OR "pré-eclâmpsia" OR "gestational diabetes" OR "diabetes gestacional" OR "birth interval" OR "intervalo entre nacimientos" OR "intervalo entre nascimentos" OR "interpregnancy interval" OR "intervalo intergenésico" OR "intervalo intergestacional" OR contraception OR anticoncepción OR contracepção OR contraceptive* OR anticonceptivo* OR contraceptivo* OR "oral contraceptive*" OR "anticonceptivos orales" OR "contraceptivos orais" OR "hormone therapy" OR "hormone replacement therapy" OR "estrogen therapy") AND ("cognitive decline" OR cognition OR dementia OR "mild cognitive impairment" OR "Alzheimer's disease" OR "deterioro cognitivo" OR "demencia" OR "declínio cognitivo") AND ("Latin America" OR "South America" OR "América Latina" OR "Sudamérica" OR "América do Sul" OR Argentina OR México OR Brasil OR Colombia OR Chile) |

**Supplementary Table 2.** *Quality assessment using the Newcastle–Ottawa Scale.* Quality assessment of the included studies using the Newcastle-Ottawa Scale. The table details the scores assigned to each study based on the criteria for participant selection, group comparability, and outcome assessment, providing an overview of the methodological quality of the reviewed studies.

| **Study** | **Study design** | **Selection** | **Comparability** | **Outcome/Exposure** | **Total (Quality)** |
| --- | --- | --- | --- | --- | --- |
| Monterrosa-Castro Á. et al. | Cross-sectional | ★★ | ★★ | ★★★ | 7 (Good) |
| Blümel J.E. et al. | Cross-sectional | ★★ | ★★ | ★★ | 6 (Satisfactory) |
| Vallejo M.S. et al. | Cross-sectional | ★★★ | ★★ | ★★ | 7 (Good) |
| Espinoza M.T. et al. | Cross-sectional | ★★ | ★★ | ★★ | 6 (Satisfactory) |
| Barbosa et al. | Cross-sectional | ★★★ | ★★ | ★★ | 7 (Good) |
| Calle A. et al. | Cross-sectional | ★★ | ★★ | ★★ | 6 (Satisfactory) |
| Blümel J.E. et al. | Cross-sectional | ★★ | ★★ | ★★ | 6 (Satisfactory) |
| Matzumura-Kasano J.P. et al. | Cross-sectional | ★★ | ★★ | ★★ | 6 (Satisfactory) |
| Cortés Y.I. et al. | Cross-sectional | ★★★ | ★★ | ★★ | 7 (Good) |
| Stickel A.M. et al. | Longitudinal | ★★★ | ★★ | ★★ | 7 (Good) |
| Blümel J.E. et al. | Case-control | ★★ | ★★ | ★★ | 6 (Satisfactory) |
| Pérez Sampayo et al. | Cross-sectional | ★★★ | ★★ | ★★ | 7 (Good) |
| Carranza-Lira | Cross-sectional | ★★ | ★★ | ★★ | 6 (Satisfactory) |
| Prince M.J. et al. | Longitudinal | ★★★ | ★★ | ★★ | 7 (Good) |
| Zilberman J.M. et al. | Cross-sectional | ★★ | ★ | ★★ | 5 (Satisfactory) |
| Fernandes R.C.L. et al. | Cross-sectional | ★★ | ★ | ★★ | 5 (Satisfactory) |
| Pereira F.P. et al. | Longitudinal | ★★ | ★ | ★★ | 5 (Satisfactory) |
| Hómez de Delgado. et al. | Longitudinal | ★★ | ★ | ★★ | 5 (Satisfactory) |
